# Performance of novel enamel-conditioning calcium-phosphate pastes for orthodontic bonding: An *in vitro* study

**DOI:** 10.4317/jced.60028

**Published:** 2023-02-01

**Authors:** Hayder A. Kadhim, Sanjukta Deb, Ali I. Ibrahim

**Affiliations:** 1 Department of Orthodontics, College of Dentistry, University of Baghdad, Baghdad, Iraq; 2 Department of P.O.P., College of Dentistry, University of Kufa, Najaf, Iraq; 3 Centre for Oral, Clinical and Translational Sciences, Faculty of Dentistry, Oral & Craniofacial Sciences, King’s College London, London, UK

## Abstract

**Background:**

This study aimed to develop remineralizing calcium-phosphate (CaP) etchant pastes for enamel conditioning before bracket bonding and evaluate the bonding performance, failure pattern, and enamel surface integrity post bracket debonding in comparison with the conventional phosphoric acid (PA) etchant gel.

**Material and Methods:**

Micro-sized monocalcium phosphate monohydrate and hydroxyapatite (micro- and nano-sized) powders were mixed with various phosphoric and nitric acid concentrations to develop eight acidic CaP pastes. Ninety extracted human premolars were randomly assigned into eight experimental and one control group (n=10). The developed pastes and control (commercial 37% PA-gel) were applied onto the enamel using the etch-and-rinse protocol before bonding metal brackets. Shear bond strength and adhesive remnant index (ARI) scores were evaluated after 24 hours water storage (24 h) and post 5000 thermocycling (TC). Field emission scanning electron microscopy (FE-SEM) was used to evaluate enamel damage after bracket debonding.

**Results:**

The developed CaP pastes, excepting MNA1 and MPA1, resulted in significantly lower SBS values and ARI scores than 37% PA gel. Etching with 37% PA yielded roughened, cracked enamel surfaces with excessive retention of adhesive residue. In contrast, enamel treatment with the experimental pastes exhibited smooth, unblemished surfaces, with obvious CaP re-precipitation induced by mHPA2 and nHPA2 pastes and to a lesser extent by MPA2 paste.

**Conclusions:**

Three newly developed CaP etchant pastes (MPA2, mHPA2, and nHPA2) can be promising alternative enamel conditioners that outperform conventional PA by generating adequate bracket bond strengths besides precipitating CaP crystals on the enamel. Moreover, these pastes maintained unblemished enamel surfaces with no or minimal adhesive residue after bracket removal.

** Key words:**Enamel Conditioning, Calcium Phosphate, Bracket Bond Strength, Orthodontic Bonding, enamel damage.

## Introduction

Reliable adhesion between fixed orthodontic attachments and enamel surface is a key factor for the clinical success of orthodontic treatment (1). Bracket bonding using the etch-and-rinse technique, which involves enamel pre-treatment with 37% PA, is the most commonly used practice by orthodontists as it ensures secure attachment of the bracket to enamel surface. However, routine etching with PA irreversibly removes several microns of the enamel surface and induces demineralization, which renders the enamel susceptible to acid attacks and caries, especially around orthodontic attachments (2). In addition, the resultant high bond strength causes a wide range of enamel damage upon bracket debonding (3) with excessive adhesive residues left on enamel that necessitate a prolonged chair time for removal using traumatic rotary burs, which have been shown to induce inevitable enamel scratching (4). The use of alternative acids to PA for enamel conditioning before orthodontic bonding has been attempted in the literature (5); of these, nitric acid was reported to produce bond strengths comparable to phosphoric acid. However, this acid demonstrated almost identical extent of etch and erosion to PA, with considerable loss of natural tissue morphology and similar incidence of enamel damage upon bracket debonding (6,7).

On the other hand, alternative strategies were sought to minimize these inconsistences such as the use of self-etch primers and laser irradiation. Self-etch primers introduced as simpler, time-conserving conditioning technique and exhibited bracket bond strength effective for clinical use. However, a conflicting data were reported with the respect to incidence of enamel damage upon bracket removal (8). Laser irradiation was suggested as an effective conditioning modality to roughen the tooth surface and yielded bond strengths comparable or even higher than acid etching. However, it was associated with excessive surface roughness, cracking with more adhesive remnants left on enamel surface post debracketing (9). Other approaches involved the development of orthodontic adhesives containing various calcium phosphate particles with different concentrations to release supersaturated level of Ca and *P* ions onto the enamel surface, thus minimizing demineralization. However, to ensure long-term ions release, they require repeated immersion in Ca and *P* ions-rich solutions for recharging. In addition, the failure mode at bracket debonding has mostly occurred at the bracket-adhesive interface, leaving excessive remnant adhesive on the tooth surface. Moreover, a reduction in the bracket bond strength was reported after 1-month of storage in aging solutions (10,11).

In contrast to the traditional concept of CaP ion release, a novel etchant system was introduced adopting a new concept: enamel conditioning concomitant with CaP precipitation before orthodontic bonding. An acidic CaP paste was developed by incorporating two CaP compounds with phosphoric acid solution, capable of producing a gentler etch pattern than conventional PA, clinically effective bond strengths, with no or minimal adhesive residue and un-damaged enamel upon bracket debonding. However, preparation of the etchant paste entailed a difficult manipulation via manual mixing of two types of CaPs, basic (β-TCP) and acidic (MCPM) powders, with 37% PA on a glass slab for 30 seconds before application (12). Therefore, this pilot study aimed at developing a CaP-based conditioning system in a simplified and more clinically-convenient form (capsule formulation) for enamel etching before bracket bonding by mixing one CaP compound with an acidic solution. The hypothesis is that the developed etchant system could outperform conventional 37% PA in minimizing adhesive remnants, maintaining the integrity and possibly remineralizing the enamel surface via CaP precipitation without undermining the bracket bond strength. Performance was assessed by three parameters: 1) shear bond strength, 2) incidence of enamel damage, and 3) the residual adhesive left on the enamel.

## Material and Methods

Ninety premolars were recruited for this study after acquiring the ethical approval (Ref. no.: 355421/355). The teeth were collected from 15-30 years-old patients requiring extraction before orthodontic treatment. The buccal surfaces of teeth were examined using a digital microscope (Dino-lite pro, AnMo Electronics Co., Taiwan) at x20 magnification. Teeth with malformations, cracks, fractures, history of orthodontic or bleaching treatment, caries and restoration were excluded. After extraction, the teeth were cleaned and stored in 1% chloramine-T trihydrate as a bacteriostatic/bactericidal solution for one week, followed by storage in distilled water until the time of bracket bonding (ISO/TS 11405:2015).

Each tooth was embedded vertically in an acrylic block using a rubber mold (18x18x15 mm). Using the analyzing rod of a surveyor, the buccal surface of the crown was aligned such that bracket base parallel to the debonding force applied by a chisel during the shear bond test. Self-cure acrylic resin (Major Repair, Major Prodotti Dentari S.P.A., Italy) was poured around the tooth up to about 1 mm apical to the level of cemento-enamel junction. The mounted teeth were randomly allocated into 9 groups (n=10), and stored in distilled water (DW) at lab temperature until bonding time.

-Formulation of experimental etchant pastes 

Nano- and micro-sized Hydroxyapatite (HA) and micro-sized monocalcium phosphate monohydrate (MCPM) powders (Sigma-Aldrich CHEMIE GmbH Co., Steinheim, Germany) were mixed with various concentrations of phosphoric acid (PA) and nitric acid (NA) to develop the experimental CaP pastes. PA solutions of 2%, 5%, 37%, and 40% (by weight) were prepared by diluting PA (85 wt%, CARLO ERBA Reagent, France) with distilled water (DW). In addition, NA solutions of 0.5% and 1% concentrations were prepared by diluting NA (65 wt%, Riedel-de Ha¨en, Sigma–Aldrich Laborchemikalien GmbH, Seelze, Germany) with DW.

Eight experimental CaP pastes were prepared as follows.

**Figure 1:**
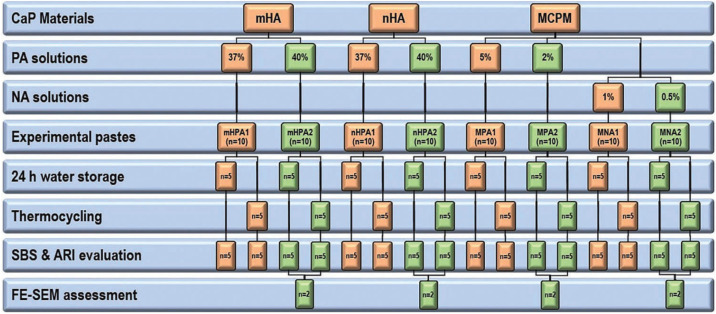
A flowchart depicting the preparation and testing of experimental CaP pastes.

Nano- and micro-sized HA powders were mixed with 37% and 40% PA solutions in a 0.4:1 powder-to-liquid ratio to formulate nHPA1, nHPA2, mHPA1 and mHPA2 pastes, respectively. Micro-sized MCPM powder was mixed with 2% and 5% PA solutions in a 6:1 powder: liquid ratio to prepare MPA1 and MPA2 etchant pastes, respectively. Similar to the latter ratio, MCPM powder was mixed with 1% and 0.5% NA solutions to formulate MNA1 and MNA2 pastes (Fig. 1).

The assigned quantity of powder and liquid of each formulation was added into a 2ml Eppendorf tube and placed in a dental amalgamator (Lingchen dental Co., Ltd, China) as a “mixing capsule”. After 15 s of mixing, a homogenous workable paste was obtained and ready to be applied to the buccal surface of the tooth. The pH of the resultant pastes was immediately assessed using a digital pH meter supplied with a flat-surface electrode (Model S450CD, Sensorex, USA). The mixing procedure and pH measurement were conducted under ambient lab conditions (22-25°C, 30-40% humidity).

-Enamel conditioning and bracket bonding

The buccal surface of the crown of each tooth was polished with oil-free pumice using rubber cups at a low speed for 10 s. The conventional etch-and-rinse technique was used for enamel conditioning of all groups, using 37% PA gel (Proclinic, Madrid, Spain) for the control group, and CaP pastes for the experimental groups; the etchant was applied onto the middle third of buccal surface for 30 s then washed (10 s) and dried (10 s). Stainless steel premolar brackets (MBT prescription with 0.022” slot, Pinnacle, Orthotechnology, USA) were bonded using Transbond XT primer and adhesive (3M Unitek, Monrovia, California, USA).

A thin layer of Transbond XT Primer was applied on the etched enamel, then each bracket base was loaded with a thin layer of Transbond XT adhesive and positioned on the center of the buccal surface with 300 g firm pressure for 10 s (Dontrix force gauge, DTC Medical Apparatus Co., Hangzhou, China). Excessive adhesive around the bracket was removed, followed by polymerization from two sides of the bracket (mesial and distal, 10 s each) using LED Curing Light (SDI Radii Plus, 1,500mw/cm2 light intensity, Victoria, Australia). The specimens were kept in distilled water at 37°C for 24 hours, then half of the specimens (n=5) of each group was exposed to the bond strength testing, while the other half was subjected to 5000 cycles of TC before measuring the debonding force. Each complete cycle lasted for 65 s: 30 s dwelling time in each water bath, and 5 s transfer time.

-Measurement of SBS and adhesive remnant index (ARI)

Bracket debonding was conducted to measure the SBS using a chisel on an electronic Universal Testing Machine (WDW-100E, Time Group Inc., Beijing, China) applied vertically to the bracket base in an occluso-gingival direction with a crosshead speed of 0.5 mm/min at 4 KN load setting. The resultant debonding force (in Newtons) was divided by the bracket surface area (11.46mm) to calculate SBS in megapascal (MPa). After bracket debonding, the tooth surface was observed under a digital microscope at x20 magnification to examine enamel damage (crack or tear-out), and assess the remnant adhesive on the enamel scored according to the ARI system (13): 0): No adhesive left on the tooth, 1): Less than half of the adhesive left on the tooth, 2): More than half of the adhesive left on the tooth, 3): All adhesive left on the tooth with a distinct impression of the bracket mesh.

-FE-SEM assessment of de-bracketed enamel surface

FE-SEM evaluation of de-bracketed enamel surfaces was conducted for the experimental CaP pastes that survived the TC regime (MPA2, MNA2, mHPA2, and nHPA2) and compared with the control group. Two specimens of each group were randomly selected, the crown was cut longitudinally across the central fossa using a diamond wafering blade to obtain a de-bracketed buccal surface. The samples were kept dry for 24 hr at ambient lab conditions, then sputter-coated 

with gold-palladium particles (15 nm) before examination with FE-SEM machine (FEI Inspect S50, Czech Republic) at an accelerating voltage of 20 kV. The enamel surface was evaluated according to the enamel damage index (14) as follow:

Grade 0: Smooth surface without scratches, and perikymata might be visible.

Grade 1: Acceptable surface, with fine scattered scratches.

Grade 2: Rough surface, with numerous coarse scratches or slight grooves visible.

Grade 3: Surface with coarse scratches, wide grooves, and enamel damage visible to the naked eye.

-Statistical analysis 

SPSS Statistical software (Version 25.0, IBM Corp., Armonk, USA) was used for statistical analyses. The data showed normal distribution (Shapiro-Wilk test, *p*> 0.05), and the assumption of homogeneity of groups’ variances was not violated (Levene test, *p*> 0.05). The difference in SBS with regard to etchant material and testing time was assessed using a two-way analysis of variance (ANOVA), followed by Dunnett post-hoc test to determine which experimental group differ significantly from the control. The statistical significance of the difference in ARI scores were analyzed using Mann-Whitney test. All data comparisons were conducted at a 0.05 significance level.

## Results

Results of SBS and pH values are presented in Table 1. ANOVA test indicated a significant difference between the groups regarding etchant material (*p*<0.001), and testing time (*p*=0.012). The Dunnett test demonstrated that all experimental pastes had significantly lower SBS values than the control (*p*<0.05), except MNA1 and MPA1 pastes which showed a comparable bond strength to 37% PA gel. However, the TC protocol did not adversely influence SBS in all groups, except the mHPA1 and nHPA1 groups where the SBS was significantly reduced.

**Table 1:**
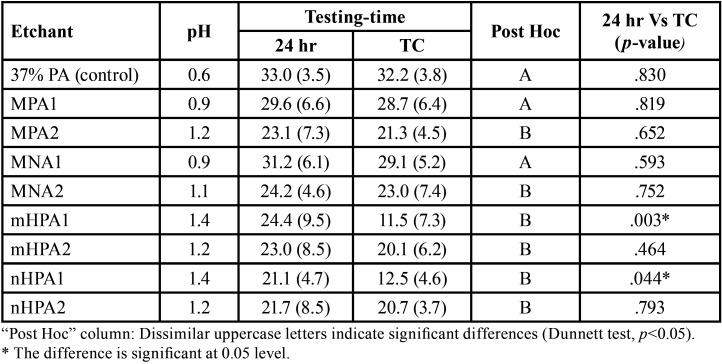
Descriptive and inferential statistics of SBS (MPa).

Regarding the ARI scores, statistically significant differences were found between the experimental CaP pastes and control, except for MPA1 and MNA1 groups that showed comparable ARI scores to the 37% PA gel (Table 2). Specimens treated with 37% PA and two experimental CaP pastes (MPA1 and MNA1) retained greater amounts of adhesive remnants (mainly scores 2 and 3), while enamel etching with other experimental pastes resulted in significantly less adhesive residues on the tooth surface (mostly scores0 and 1) post bracket debonding.

**Table 2:**
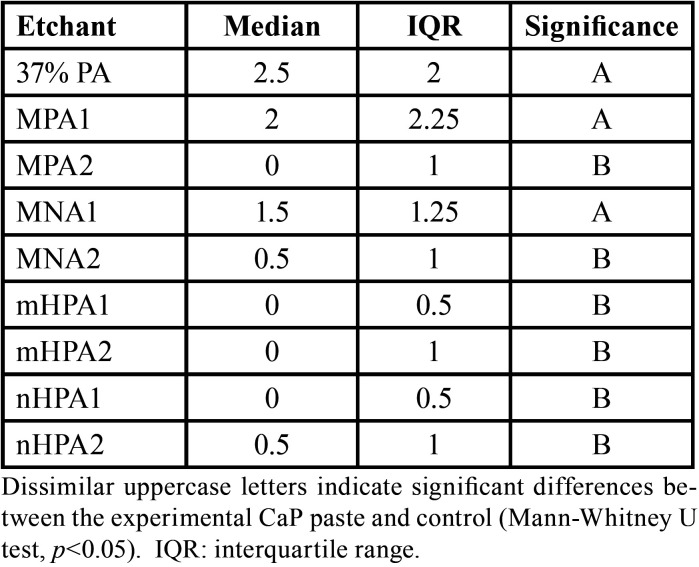
Descriptive and inferential statistics of ARI scores.

**Figure 2:**
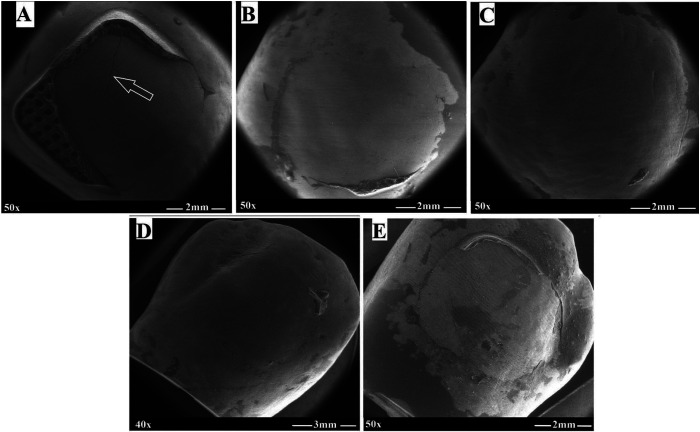
FE-SEM macrographs of debonded premolar buccal surfaces. (A) 37% PA, (B) MNA2, (C) MPA2, (D) mHPA2, and (E) nHPA2.

**Figure 3:**
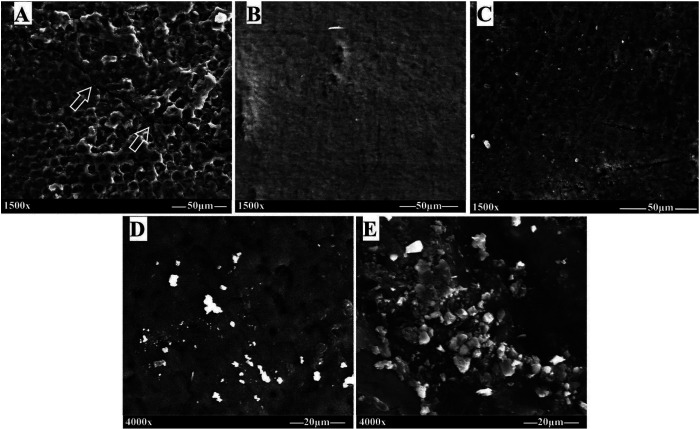
FE-SEM micrographs of de-bracketed buccal surfaces. (A) 37% PA, (B) MNA2, (C) MPA2, (D) mHPA2, and (E) nHPA2.(D) mHPA2, and (E) nHPA2.

FE-SEM macrographs and micrographs of debracketed buccal surfaces are presented in Figures 2 and 3, respectively. The 37% PA group revealed signs of enamel damage (cracks) with retention of adhesive resin (Fig. 2A), whilst the experimental CaP groups demonstrated smooth, un-damaged surfaces with preservation of perikymata, and no or minimal adhesive residues (Fig. 2B-E). With respect to EDI, enamel etching with 37% PA produced a mixture of grades 2 and 3, a rough cracked surface with coarse scratches (Fig. 3A), whilst CaP pastes yielded a mixture of grades 0 and 1, generally smooth, regular surfaces with dispersed micropores and few fine scratches (Fig. 3B-E). Moreover, CaP precipitants were noticed on surfaces treated with mHPA2, nHPA2 pastes (Fig. 3D,E) and to a lesser extent with MPA2 paste (Fig. 3C), which were not seen on MNA2-treated surfaces (Fig. 3B).

## Discussion

The enamel is often at a high risk of demineralization and development of WSLs during orthodontic treatment, especially when oral hygiene habits are doubtful. In addition, with fixed orthodontic therapy, the enamel surface is routinely etched with phosphoric acid that produces the appropriate etching pattern for a robust bracket-enamel adhesion to preclude bracket failure throughout the treatment period (10). Conventional enamel treatment with PA is not an enamel-friendly approach because of the extensive and profound demineralization that can reduce the mechanical properties (15), and the excessively high resultant bond strength induces enamel cracks or tear-out upon bracket debonding (16). Therefore, the enamel-conditioning technique needs to be optimized to establish clinically useful bond strengths while minimizing the debilitating effect at etching process and damage incidence upon bracket removal. In the present study, three newly-developed CaP pastes (MPA2, mHAP2 and nHPA2) showed a dual conditioning/CaP-precipitating effect on the enamel surface, and provided bond strengths suitable for clinical use. The hypothesis was proven that the new CaP pastes outperform conventional 37% PA gel in maintaining the integrity and remineralizing the enamel surface via CaP precipitation without compromising the bracket bond strength.

The SBS findings revealed significantly higher mean SBS values for 37% PA, MPA1 and MNA1 groups than other experimental groups, which can be attributed to the strong etching effect of 37% PA gel, MPA1 and MNA1 pastes that is relevant to their low pH values: 0.6, 0.9 and 0.9, respectively. It was reported that the etchants with lower pH values possess greater acidity, hence causing more pronounced etching patterns (8). During the application of such etchants, the pH remains most likely unaffected by the dissolution of HA from the enamel surface resulting in a pronounced etching effect with prominent inter-prismatic conditioning and rough enamel surfaces (17). Bonding the brackets to such surfaces results in thick resin tags that deeply penetrate the enamel substrate, yielding high bond strengths (18). Accordingly, etchant materials with a relatively less acidic pH show lower conditioning potential and produce ill-defined etch-patterns with mild surface roughness, leading to lower bond strengths (19). This can account for the lower SBS means recorded by other CaP experimental pastes with higher pH values in the range of 1.1-1.4. The use of low concentrations of PA and NA besides mixing of HA powders with the high PA concentrations effectively lowered the acidity of resultant experimental pastes. With respect to MPA2 and MNA2 pastes, reduced PA (2%) and NA (0.5%) concentrations were mixed with MCPM microparticles in a 6:1 ratio. On the other hand, for the experimental pastes: mHPA1, mHPA2, nHPA1 and nHPA2, the dissolution of the HA particles (micro- and nano-sized) neutralized PA solutions of high concentrations (37% and 40%) by raising the pH of resultant etchant pastes. Thus, when these pastes were applied on the tooth surface, a conservative demineralization process might occur producing a milder etching effect which in turn yields a relatively lower bond strength (19,20). However, the recorded SBS values were above 6-10 MPa which was proposed as a threshold for clinical demand (21), hence these values being suiTable for successful clinical performance.

The specimens of mHPA1 and nHPA1 groups exclusively demonstrated a significant drop in SBS values following TC protocol, in addition to failure of one bracket in each group during shear bond test. This can be traced back to the relatively high pH value (1.4) of these pastes, which generated a more conservative etch-pattern allowing for a shallower resin penetration that could not survive the aggressive thermal stresses and frequent exposure to water during TC. It was reported that these stresses with water absorption and solubility of the composite can significantly influence bracket adhesion, resulting in reduced bond strengths or even bond failure (22). Furthermore, the TC procedure induces mechanical stresses relevant to differential thermal changes, which can directly promote crack propagation in enamel-adhesive-bracket interfaces (16).

The ARI findings demonstrated that etchant type had a significant effect on the mode of bracket failure, yielding various sites of bond failure according to the etchant used. The most frequent mode of failure was an adhesive failure at the bracket/adhesive interface in the control, MPA1 and MNA1 groups, leaving the majority of adhesive on the tooth surface (ARI scores 2 and 3); this implied that the bond strength was greatest at the enamel/adhesive interface. Similar findings were observed when enamel was etched using an etchant of strong acidity such as 37% PA, where an intense interaction occurs with the enamel surface producing an ideal retentive morphology for adhesive material, hence more resin adhesive remains on the enamel surface upon bracket debonding (18). This would necessitate considerable chair time to remove the adhesive residue with the added chance of hurting the enamel surface during the cleaning process (4). In contrast, other experimental pastes revealed a prevalence of failure at the enamel/adhesive interface recording significantly lower ARI scores (mostly 0 and 1). Modifying the composition and the pH of experimental etchant pastes probably played a significant role in the conservative interaction with enamel, hence promoting clinically desirable failure mode at the enamel/adhesive interface that makes the debonding and subsequent polishing much easier (21). This failure mode was frequently associated with enamel etchants of relatively low acidity that induce a milder etch-pattern (23).

It was reported that the greater bond strength often associated with a concomitant increase in the risk of enamel damage (8,16). The FESEM observations of enamel surfaces etched with 37% PA gel came in accordance with the reported data, exhibiting retention of adhesive resin with enamel cracking and tear-out seen on several specimens post debonding (Fig. 2A,3A). Moreover, the control group showed markedly roughened enamel surfaces with coarse scratching, high grades of EDI (grade 2 and 3), and deteriorated mechanical properties of enamel structure due to the high demineralization potential. This pattern besides the deeply penetrated thick resin tags render the enamel more liable for cracking and fracturing during debracketing (24). On the other hand, enamel conditioning with the experimental CaP pastes, namely MNA2, MPA2, mHPA2, and nHPA2 (Fig. 2B-E, 3B-E) left smooth, unblemished enamel surface, retaining much of the intact enamel characteristics. With the experimental CaP pastes of relatively less acidity, therefore, a conservative interaction with enamel was induced, resulting in a milder demineralization effect and lower depth of resin penetration into the enamel structure, thus reducing the risk of enamel damage upon bracket debonding.

A closer view at the FESEM microscopic images elucidated precipitation of CaP particles on the enamel surfaces treated with the experimental pastes mHPA2, nHPA2, and to a lesser extent with MPA2; yet CaP precipitation could not be observed on enamel treated with MNA2 paste. Enamel acid-etching occurs as a reaction between enamel minerals (HA) and the acid molecules that terminates in HA dissolution and creation of micro-pores for mechanical retention of adhesive resin (25). The reaction products are largely dependent on the type and acidity of the etchant material. It was reported that enamel etching with PA induces HA dissolution and release of Ca and PO4 ions, which re-precipitate in the form of calcium-phosphate compounds onto the etched surface (17). Accordingly, HA powder dissolves when mixed with the aqueous solution of 40% PA, releasing calcium, phosphate, and hydroxide ions into the bulk of resultant pastes (mHAP2 and nHPA2). Simultaneously, these acidic pastes mobilize more ions from the underlying enamel when applied on the tooth surface. The abundantly released ions react and form acidic calcium-phosphate salts, which can precipitate onto the enamel surfaces etched with mHPA2 and nHPA2 experimental pastes (Fig. 3D,E). On the other hand, it was reported that CaP crystals of low solubility precipitate when etching the enamel with PA of concentrations less than 27% (26). Mixing of 2% PA with MCPM particles to formulate the MPA2 paste was enough to produce an etchant paste of low acidity that encouraged deposition of less-water soluble CaP salts when interacting with the enamel surface (Fig. 3 C). The low solubility of the CaP salts in addition to the volume of their crystallites contributed to the deposition of minerals onto the demineralized enamel surfaces even post water rinsing (27). The deposition of CaP salts may interfere with the demineralization process (17), and with the diffusion of adhesive monomers into the created surface micro-pores (12), which likely contribute to lowering the SBS values attained when conditioning the enamel with mHPA2, nHPA2, and MPA2 pastes. Furthermore, the presence of CaP crystals within the adhesive interface imposes a weakening effect that facilitates safe bond failure at the enamel-adhesive interface upon debracketing (12). Compared to PA, nitric acid is relatively a stronger mineral acid and has a lower pKa value. It was reported that nitric acid, unlike phosphoric acid, does not induce re-precipitation of CaP on the etched enamel surfaces (28). Indeed, hydroxyapatite dissolution by the nitric acid results in the formation of calcium nitrate, a hygroscopic and highly water-soluble salt, so unlikely to be precipitated after rinsing the enamel surface (29). Consequently, although MNA2 paste was shown to have comparable acidity to MPA2 paste, it could not induce CaP precipitates on the etched enamel surface due to the presence of nitric acid in its composition.

Data attained in this preliminary *in-vitro* study offer interesting alternatives to replace phosphoric acid and induce CaP precipitation concurrently with enamel conditioning, which could render the etching procedure a less damaging technique and possibly optimize orthodontic therapy, especially when using MPA2, mHPA2 and nHPA2 pastes. However, performance concerns regarding early bond efficiency and durability throughout long-term water storage and acid challenge should be addressed in future studies before proceeding with long-term clinical trials to determine the clinical efficacy of the developed conditioning system. Moreover, the impact of newly developed CaP pastes on the mechanical properties of surface enamel needs to be evaluated, particularly since these properties have been reported to be declined after phosphoric acid treatment (15).

## Conclusions

The newly developed experimental pastes MPA2, mHPA2, and nHPA2 can be promising alternative enamel conditioners capable of simultaneous etching and CaP salts precipitation on enamel surface without compromising bond strengths. Additionally, the application of these pastes consistently produced a smooth, un-damaged enamel surface, preferable enamel-adhesive bond failure with minimal or no remnant adhesive post bracket debonding.
